# TLR3 activation in astrocytes attenuates the nigrostriatal pathway degeneration in rodent models of Parkinson’s disease

**DOI:** 10.3389/fncel.2026.1746731

**Published:** 2026-02-04

**Authors:** Jaeyeong Jeong, So-Yoon Won, Young Cheul Chung, Won-Ho Shin, Byung Kwan Jin, Eun S. Park

**Affiliations:** 1 Department of Biochemistry and Molecular Biology, School of Medicine, Kyung Hee University, Seoul, Republic of Korea; 2 Department of Predictive Toxicology, Korea Institute of Toxicology, Daejeon, Republic of Korea; 3 Human and Environmental Toxicology, University of Science and Technology, Daejeon, Republic of Korea; 4 Vivian L. Smith Department of Neurosurgery, McGovern Medical School, The University of Texas Health Science Center at Houston, Houston, TX, United States; 5 Center for Neuroimmunology and Glial Biology, The Brown Foundation Institute of Molecular Medicine, The University of Texas Health Science Center at Houston, Houston, TX, United States

**Keywords:** astrocytes, dopamine neuron, neurotrophic factors, Parkinson’s disease, poly I: C, toll-like receptor 3

## Abstract

Toll-like receptor 3 (TLR3) is classically known for mediating inflammatory pathways in Parkinson’s disease (PD). However, the role of TLR3 in nigrostriatal degeneration in PD remains unclear. Here, we observed that TLR3 is predominantly expressed on astrocytes in the substantia nigra in both human PD brain and in rat PD models induced by intra-MFB injection of 1-methyl-4-phenylpyridinium (MPP^+^). Interestingly, Poly I: C, an activator of TLR3, significantly induced TLR3 expression on astrocytes. Treatment with Poly I: C markedly attenuated nigral dopamine neuron death in the PD rat models. The survival of dopamine neurons was accompanied by the production of ciliary neurotrophic factor and vascular endothelial growth factor-B on astrocytes in Poly I: C-treated PD rats. The attenuation of dopamine neuron death was also observed in the Poly I: C-treated AAV2-hα-syn-A53T-induced rat PD model. Our findings suggest that activating TLR3 in astrocytes could be a potential therapeutic strategy for attenuating PD progression.

## Introduction

Parkinson’s disease (PD) is a progressive neurodegenerative disease with early prominent degeneration of dopamine (DA) neurons in the substantia nigra pars compacta (SNpc) and DA deficiency in the striatum (STR), which leads to behavioral deficits (Braak et al., 2004; Dauer and Przedborski, 2003). Accumulating evidence displayed that the progression of sporadic PD caused by neurotoxin (1-methyl-4-phenylpyridinium, MPP^+^) exposures and α-synuclein (α-Syn) is accompanied by death of DA neurons and loss of striatal fibers (Kohutnicka et al., 1998; Yun et al., 2018; Park et al., 2012). Given these detrimental factors as potential causes of PD etiology, extensive work has been undertaken to reverse or halt the progression of DA neuron loss.

Toll-like receptor 3 (TLR3) plays a key role in the innate immune response (Negishi et al., 2008). TLR3 has been detected in diverse cell types, including neurons, microglia, astrocytes, and myeloid dendritic cells (Prehaud et al., 2005; Town et al., 2006; Farina et al., 2005; Jongbloed et al., 2010). Endogenous TLR3 is activated by treatment with Polyinosinic-polycytidylic acid (poly I: C), a synthetic TLR3 agonist, but the effect of poly I: C on the progression of PD remains controversial. The intranigral injection of poly I: C induced microglia/astrocyte activation in the SNpc and increased vulnerability to nigral neuron death in intra-striatal-6-hydroxydopamine (6-OHDA)-injected rats (Deleidi et al., 2010). In contrast, another study showed that the pretreatment of poly I: C markedly showed a neuroprotective effect by reducing infarct volume and attenuating inflammation in the ischemic stroke mouse model (Pan et al., 2012; Packard et al., 2012). The diverse role of TLR3 in the cellular context of PD pathology still needs to be identified.

Direct delivery of neurotrophic factors (NTFs) or boosting endogenous levels of NTFs in the brain have been shown to protect DA neurons (Sullivan and O'Keeffe, 2016; Sampaio et al., 2017; Chmielarz and Saarma, 2020). Astrocytes are the abundant glial cells in the mammalian brain and play crucial roles in regulating brain homeostasis (Sofroniew and Vinters, 2010; Kam et al., 2020; Miyazaki and Asanuma, 2020). In post-mortem brains of PD patients, reactive astrocytes have been identified, which can produce NTFs under pathological conditions and lead to neuronal survival (Miklossy et al., 2006; Liddelow et al., 2017; Patel et al., 2019; Poyhonen et al., 2019; Duarte Azevedo et al., 2020). Notably, astrocytes express various NTF, including ciliary neurotrophic factor (CNTF) or vascular endothelial growth factor B (VEGF-B) that attenuate or rescue the death of DA neurons in experimental rodent or culture PD models (Muller et al., 2007; Rothhammer et al., 2018; Nam et al., 2015; Yue et al., 2014; Falk et al., 2009). Surprisingly, in human astrocytes, Poly I: C-induced TLR3 activation enhances the expression of NTFs, including CNTF (Bsibsi et al., 2006; Li et al., 2012). Based on this knowledge, exploring the role of TLR3 on NTF production using an experimental rodent PD model will open a new spectrum to investigate the therapeutic strategy for treating PD patients.

In the current study, we investigated the role of TLR3 in astrocytes using a PD rat model displaying degeneration of nigral DA neurons and striatal fibers caused by neurotoxin (MPP^+^). Using a Poly I: C, a TLR3 agonist, we revealed that TLR3 activation attenuates progressive neurodegeneration in PD rats. Moreover, we showed that TLR3 activation attenuates the death of nigral DA neurons in rats injected with an adenovirus-associated virus (AAV) carrying the A53T mutant α-Syn. The clinical relevance of our study was supported by demonstrating TLR3 expression in astrocytes in the human PD brain. Together, our results demonstrate that the astrocytic TLR3 is required to attenuate PD pathogenesis.

## Method

### Animals and ethical approval

The care of animals and the experiments were conducted in accordance with the guidelines approved by the Committee on Animal Research of Kyung Hee University (KHU-ASP-20-235; 30 June 2020). Female Sprague–Dawley rats (10 weeks of age, weighing 240–270 g), obtained from Daehan Biolink (originally from Taconic Co., Albany, NY, United States), were housed in temperature-controlled (21–23 °C) and humidity-controlled conditions with a 12:12-h light/dark cycle. The rats for the control or experimental groups were separated and housed until they were sacrificed. During the animal housing and experimental periods, all animals had unrestricted access to food and water. All experiments were conducted with the aim of minimizing animal suffering and utilizing the minimum number of animals required to generate meaningful scientific data.

### Animal models

Stereotaxic surgery was performed according to previously described procedures (Park et al., 2012). Rats were anesthetized through intraperitoneal injection of chloral hydrate (360 mg/kg; Millipore Sigma, St. Louis, MO, United States) and then secured in a stereotaxic apparatus (David Kopf Instrument, Tujunga, CA, United States).

#### Neurotoxin-induced PD model

According to the rat brain atlas, rats received a unilateral injection of 7.4 μg of 1-methyl-4-phenylpyridinium (MPP^+^) (Millipore Sigma, St. Louis, MO, United States) dissolved in 2 μL of phosphate-buffered saline (PBS, Gibco, Palsey, UK) into the right medial forebrain bundle (MFB) at coordinates 3.6 mm posterior to bregma, 2.0 mm lateral to the midline, and 7.5 mm beneath the skull of the brain. The injections were performed using 30-gage Hamilton syringes (Hamilton Company, Reno, NV, United States) at a rate of 0.2 μL/min, as previously described (Park et al., 2012).

#### Genetic trigger (AAV2-α-Syn-A53T)-induced PD model

To establish a genetically mimicked PD model, we used recombinant adeno-associated virus serotype 2 (AAV2) containing the A53T mutated human SNCA gene (AAV2-α-synuclein-A53T; α-syn) or AAV2-eGFP as a control. The original creator, Dr. Hiroyasu Sato, generously donated the viral vectors that express the human A53T α-syn under the control of cytomegalovirus enhancer/chicken β-actin (CAG) promoter (Sato et al., 2011; Niwa et al., 1991). Those expression and packaging vectors were co-transfected into HEK 293 T cells using jetPEI (Polyplus, Illkirch-Graffenstaden, France). The produced viruses were harvested 72 h after transfection. The titer of the viral vector is 1×10^14^ GC/ml (KIST virus facility, Seoul, South Korea). Rats received unilateral injection of 2 μL AAV2-*α*-syn-A53T or AAV2-eGFP into the right substantia nigra (SNpc; 5.3 mm posterior to bregma, 2.3 mm lateral to the midline, 7.6 mm beneath the skull of the brain). Respective controls for each injection (shCtrl) were administered at equivalent coordinates.

#### Polyinosinic-polycytidylic acid treatment

Polyinosinic-polycytidylic acid (poly I: C; obtained from Invivogen, San Diego, CA, United States) or vehicle (physiological water; Invivogen) was stereotaxically injected at a rate of 0.2 μL per minute into the right SNpc at coordinates of 5.3 mm posterior to bregma, 2.3 mm lateral to the midline, and 7.6 mm beneath the skull of the brain. Given a prior report demonstrating neuroinflammatory responses resulting in increased vulnerability of DA neurons with 10 μg of poly I: C, we conducted pilot studies with three doses (2, 4, and 10 μg) to determine the appropriate dosage for intranigral treatment (Deleidi et al., 2010). Poly I: C was administered either 1 week after MPP^+^ injection in MPP^+^-induced PD rat or 3 weeks after α-syn injection in AAV-α-Syn-A53T-induced PD rat.

### Amphetamine-induced rotation test

Dextroamphetamine (2.5 mg/kg, intraperitoneally; US Pharmacopeia, Bethesda, MD, United States) was used to induce ipsilateral rotation behavior in rats that were unilaterally lesioned in the nigrostriatal pathway by MPP^+^, as described previously (Nam et al., 2015; Kim et al., 2021). Ipsilateral rotations were counted for 1 h at 1 and 2 weeks after MPP^+^ by Ethovision video analysis software (Noldus, Wageningen, Netherlands). The absolute value of the number of rotations can indicate disease severity; however, it is highly variable across samples. Therefore, we assessed statistical significance within groups by comparing the before and after values for poly I: C or Vehicle treatment.

### Tissue preparation and immunohistochemical staining for human PD brain and rat PD model

Human brain tissue was acquired from the Victoria Brain Bank Network (VBBN) located in Parkville, Victoria, Australia. Details on control subjects and PD patients are provided in Supplementary Table S1. The human tissues were deparaffinized in xylene and subjected to citrate antigen retrieval prior to immunohistochemistry. Briefly, brain tissues were boiled in an antigen retrieval buffer containing 10 mM sodium citrate for 20 min. And tissues were chilled and washed with PBS containing 0.5% BSA. After that, tissues were blocked for 1 h in PBS containing 1% BSA, 0.1% cold fish gelatin, 0.5% Triton X-100, 0.05% Tween-20, and 0.05% sodium azide. The procedures and materials used are the same as the rat brain immunostaining protocol.

PD rats were transcardially perfused with physiological saline containing 0.5% sodium nitrate and heparin (10 U/mL) and fixed with 4% paraformaldehyde in 0.1 M phosphate buffer (pH 7.4). After dehydration in 30% sucrose solution, rat brains were coronally cut into 40-μm-thick slices using a sliding microtome. Serial coronal sections were processed for immunohistochemical staining using procedures previously described. Briefly, sections were rinsed in PBS and then incubated with the following primary antibodies: mouse GFAP (Millipore sigma; G3893, 1:500), mouse anti-OX-42 (CD11b; Bio-rad, Hercules, CA, United States; MCA275G, 1:300), mouse anti-TH (Abcam, Cambridge, UK; ab5423, 1:1000), rabbit anti-TH (Pel-freez, Rogers, AR, United States; P40101-150, 1:2000), rabbit anti-TLR3 (Novus Biologicals, Centennial, CO, United States; NB100-56571, 1:1000), rabbit anti-α-synuclein (phospho-S129) (abcam; ab51253, 1:2000), rabbit anti-CNTF (Santa-Cruz Biotechnology, Dallas, TX, United States; sc-13996, 1:200), rabbit anti-VEGF-B (Santa-Cruz Biotechnology; sc-13083, 1:200), and rabbit anti-GDNF (Santa-Cruz Biotechnology; sc-328, 1:400). After overnight incubation, in preparation for light microscopy, the brain tissues were incubated with the following biotinylated secondary antibodies: biotin-conjugated anti-mouse (Seracare Life Sciences Inc., Milford, MA, United States; 5260-0051, 1:400), or biotin-conjugated anti-rabbit (Vector Laboratories, Burlingame, CA, United States; BA-1000, 1:400), followed by avidin-biotin-peroxidase complex (Vector Laboratories; PK-6100, 1:1:100). The signal was detected by incubating the sections with 3,3′-diaminobenzidine tetrahydrochloride hydrate (DAB; Milliore Sigma; D5637) and viewed under a bright-field microscope (Olympus Optical, Tokyo, Japan). For fluorescent microscopy, sections were incubated with the following fluorescent-conjugated secondary antibodies: FITC-conjugated-anti-mouse (Vector Laboratories; FI-2000, 1:400), Cy3-conjugated-anti rabbit (Millipore Sigma; AP132C, 1:500), Alexa Fluor 488-conjugated-anti-mouse (Invitrogen, Waltham, MA, United States; A21200, 1:400), Alexa Fluor 594-conjugated anti-mouse (Invitrogen; A11037, 1:500) and CF405M-conjugated-anti-mouse (Biotium, Fremont, CA, United States; 20180, 1:400). The stained sections were covered with Vectashield mounting medium (Vector Laboratories), and signal was viewed under fluorescence microscopy (Carl Zeiss, Jean, Germany; LSM 700).

### Stereological estimation

To count the number of TH + neurons in SNpc, we used the optical fractionator method on a bright-field microscope (BX51, Olympus Optical) with Stereo Investigator software (MBF Bioscience, Williston, VT, United States). This unbiased stereological method of cell counting is not affected by either the reference volume (SNpc) or the size of the counted elements (neurons). In brief, draw the reason of interest (ROI), including the entire SNpc on each stained tissue section placed on the slide glass. In addition, set the grid options in the software for segmentation and draw them in the ROI. Then, count cells with positive signals on randomly selected grids, integrate the counts, and multiply by the grid number.

### ImageJ analysis

Imaging data obtained from the bright-field and confocal microscopes were analyzed as pixel intensity using the ImageJ version 1.54f (National Institutes of Health, Bethesda, MD, United States). ImageJ was used to quantify chromogenic signal intensity by thresholding the images, thereby eliminating non-specific background signal. For single-channel measurements, images were converted to 8-bit grayscale and adjusted to the threshold histogram endpoint. Then, the pixel intensity was quantified and normalized by an unstained area. The pixel intensity is the numerical value that represents the brightness of fluorescence-stained signals captured in each image. For the measurement of merged channels, the adjusted images of each channel are colocalized using a colocalization plugin. The pixel intensity of the overlaid signal is quantified. The variable, which ranged from 0 to 255 in pixel intensity, was simplified to the numerical value of the Y axis, as shown in the calculation that lowered numerical value by 1/100.

### Statistical analysis

Statistical analyses were performed using GraphPad Prism 10.3.0 (GraphPad Software, San Diego, CA, United States). Results are expressed as Mean ± Standard Deviation (SD). To determine statistical significance, Student’s unpaired *t*-test was performed between the two groups. One-way analysis of variance (ANOVA) with Turkey’s multiple comparisons test or Bonferroni multiple comparisons was performed in multiple groups. *p* < 0.05 was considered statistically significant.

## Results

### Astrocytes express TLR3 in the SNpc of the PD brain in humans and rats

**Figure 1:**
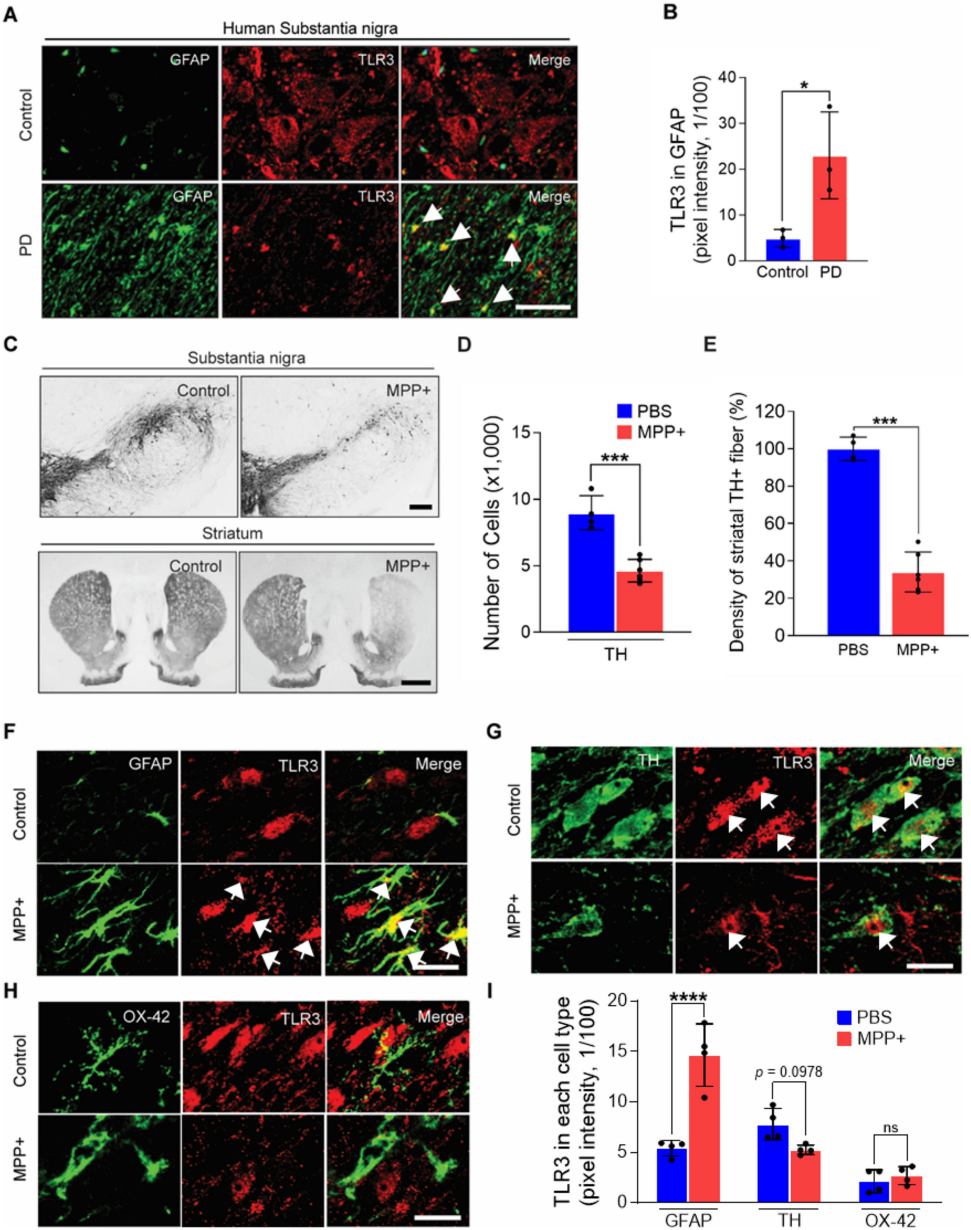
TLR3 expression in astrocytes in the SNpc of human PD and MPP^+^-induced PD rats. **(A,B)** Representative immunofluorescence images show the TLR3 expression in GFAP^+^ astrocytes in the SNpc of human control and PD brain **(A)**. Arrows indicate merged cells. Scale bars = 50 μm. Bar graph shows the quantification of TLR3 expression co-localized in GFAP^+^ astrocytes in the SNpc of the human brain **(B)**. Unpaired *t*-test. *p < 0.05. Values are means ± SD. *n* = 3 in each group. **(C–I)** Rats received a unilateral injection of MPP^+^ into the medial forebrain bundle (MFB) and were transcardially perfused at 1 week post-MPP^+^. Photomicrographs of TH^+^ cells in the Substantia nigra pars compacta (SNpc) and TH^+^ fibers in the striatum **(C)**. Scale bars = 200 μm (SNpc), 2 mm (striatum). Bar graphs show the number of TH^+^ cells in the SNpc **(D)** and optical density of TH^+^ fibers in the striatum **(E)**. Unpaired *t*-test. ***p < 0.001. Values are means ± SD. *n* = 4–6 in each group (animals). **(F–I)** Represent immunofluorescence images show the expression of TLR3 (red) in GFAP (green, **F**), or TH (green, **G**), and or OX-42 (green, **H**), and merged (yellow, arrow) in the SNpc of MPP^+^-lesioned rat brain. Scale bars = 20 μm. **(I)** Bar graph shows the quantification of TLR3 expression in each cell type. ANOVA and Turkey’s multiple comparisons test. ****p < 0.0001. ns, non-significant. Values are means ± SD. *n* = 4 in each group (animals).

We first examined TLR3 expression in SN sections obtained from human post-mortem PD patients compared to controls (Supplementary Table S1). We detected robust TLR3 immunoreactivity in GFAP immunopositive (+) astrocytes in the SNpc of PD patients compared to controls (Figures 1A,B). The expression of TLR3 was also tested in an MPP^+^-induced experimental PD rat model. Consistent with prior reports (Park et al., 2012; Nam et al., 2015; Kim et al., 2021), the unilateral injection of MPP^+^ into the median forebrain bundle (MFB) resulted in the destruction of 52% of tyrosine hydroxylase (TH) + DA neurons in the SNpc and 56% of TH + fibers in the striatum at one-week post-MPP^+^ injection compared to the PBS (as a control) (Figures 1C–E). We next determined the expression of TLR3 in glial fibrillary acidic protein (GFAP) + astrocytes, TH + DA neurons, and OX-42 (recognizes CD11b) + microglia/macrophages using immunofluorescence staining. We observed a high expression of TLR3 in GFAP+ astrocytes in the SNpc in MPP^+^-lesioned PD rats compared to the control (PBS)-injected rats (Figures 1F,I). Conversely, TLR3 expression was decreased in TH + DA neurons, while OX-42 + microglia/macrophages remained relatively unchanged (Figures 1G–I). The above data indicate that higher TLR3 expression in astrocytes in the MPP^+^-induced PD rat model is potentially relevant to human PD pathology.

### Activation of endogenous TLR3 induces the neurotrophic and neuronal survival factors in astrocytes

**Figure 2:**
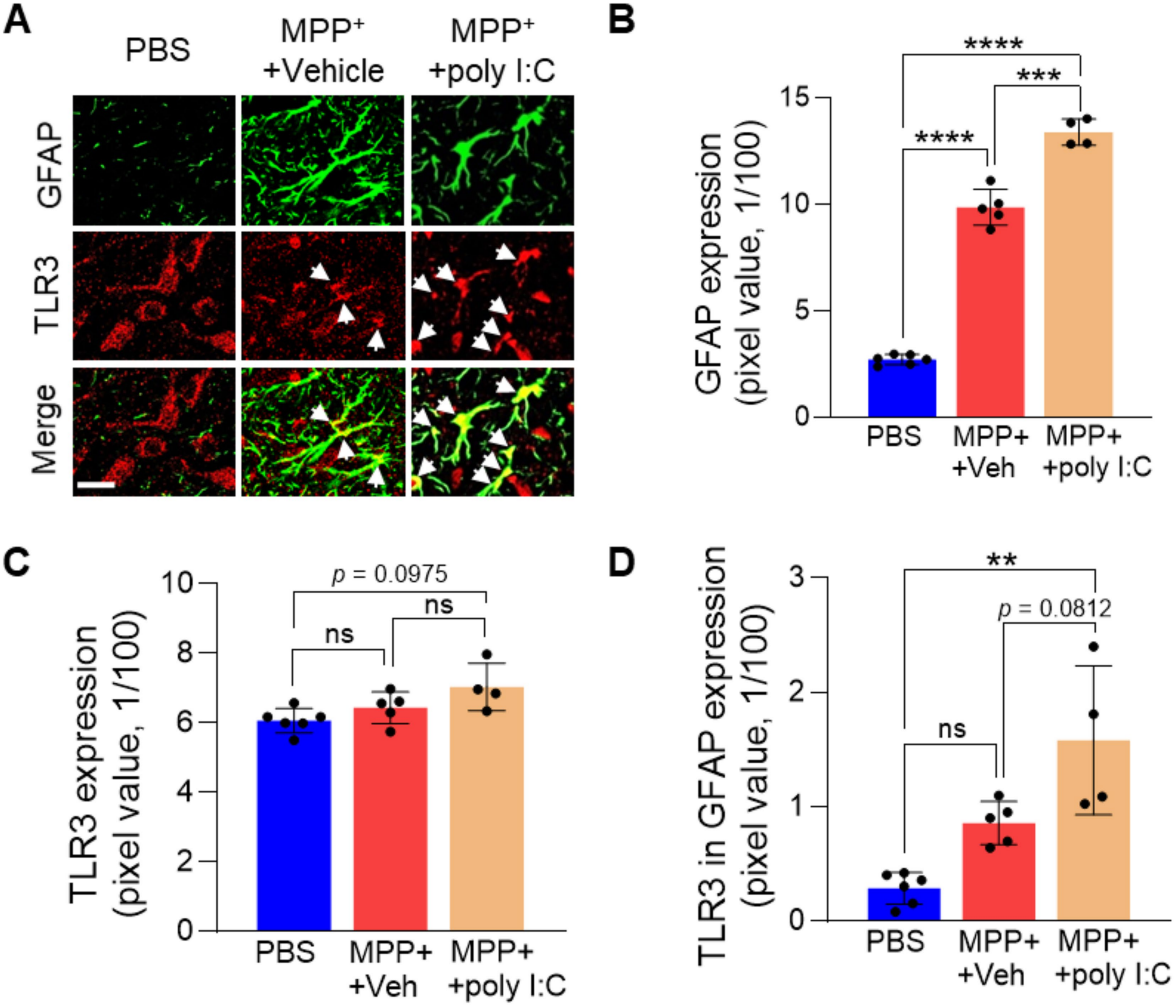
Poly I: C increases endogenous expression of TLR3 in astrocytes in the substantia nigra of MPP^+^-lesioned rats. Rats received a unilateral injection of MPP^+^ into the MFB. All rats received poly I: C (4 μg/2 μL) or vehicle intranigrally into the SNpc 7 days after MPP^+^ injection. **(A)** Representative immunofluorescence images show TLR3 expression (red) in GFAP^+^ astrocytes (green) and merged (yellow, arrow) in the SNpc of the PD rat brain. **(B–D)** Bar graph shows the quantification of GFAP **(B)**, TLR3 **(C)**, and TLR3 in GFAP^+^ astrocytes **(D)** in the SNpc of the PD rat brain. Scale bars = 20 μm. ANOVA and Turkey’s multiple comparisons test. **p < 0.01, ***p < 0.001, ****p < 0.0001., non-significant. Values are means ± SD. *n* = 46 in each group (animals).

Next, to assess the effect of endogenous TLR3 activation on MPP^+^-lesioned PD rats, the rats were treated with poly I: C, a potent TLR3 agonist. We administered 4 μg of poly I: C or vehicle into SN 1 week post MPP^+^ injection. The poly I: C dose showed no neurotoxicity, as evidenced by no significant loss of TH + DA neurons (Supplementary Figure S1). At 2 weeks, the brains were harvested, and we analyzed TLR3 activation. Interestingly, intra-nigral injection of poly I: C significantly enhanced the TLR3 immunoreactivity in astrocytes in MPP^+^-lesioned rats compared to PBS as a control for MPP^+^ and vehicle as a control for poly I: C (Figure 2).

**Figure 3:**
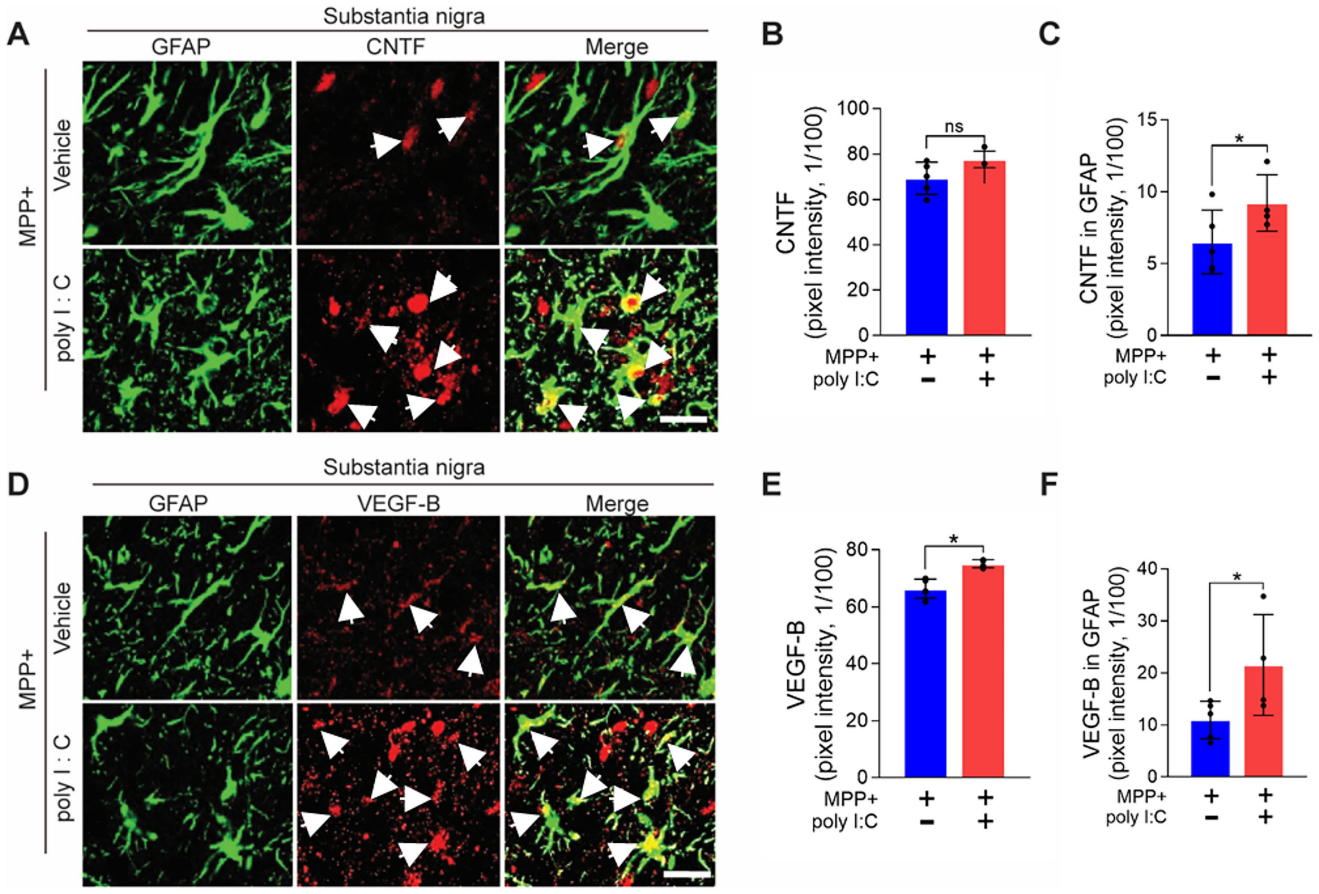
Poly I: C increases the expression of CNTF and VEGF-B on astrocytes in the substantia nigra in PD. Rats received a unilateral injection of MPP^+^ into the MFB. All rats received poly I: C (4 μg/2 μL) or vehicle intranigrally into the SNpc 7 days after MPP^+^ injection. **(A)** Representative immunofluorescence images show GFAP+ astrocytes (green) and CNTF (red) expression and merged (yellow) in the SNpc of the PD rat brain. **(B,C)** Bar graph shows the quantification of CNTF **(B)** and CNTF expression in GFAP+ astrocytes **(C)** in the SNpc of the PD rat brain. **(D)** Representative immunofluorescence images show GFAP+ astrocytes (green) and VEGF-B (red) expression, and both images are merged (yellow) in the SN of the rat brain. (**E,F**) Bar graph shows the quantification of VEGF-B **(E)** and VEGF-B expression in GFAP+ astrocytes **(F)** in the SNpc of PD rat brain. **(A,D)** Scale bars = 20 μm. **(B,C,E,F)** *n* = 4–6 in each group. Values are means ± SD. *p < 0.05. ns, non-significant. Unpaired *t*-test.

The literature showed that activated astrocytes produce neuronal survival factors, including NTF (Patel et al., 2019; Poyhonen et al., 2019; Duarte Azevedo et al., 2020). Thus, we next investigated whether endogenous TLR3 activation induces the production of neurotrophic factors in astrocytes. Immunohistochemical analysis revealed a significant increase in expression of CNTF and VEGF-B in GFAP+ astrocytes in the SNpc of poly I: C-treated MPP^+^-lesioned rat brain compared to vehicle-treated control at 2 weeks post-MPP^+^ injection (Figure 3). The data suggest that activation of endogenous TLR3 on astrocytes produces neurotrophic and neuronal survival factors.

### Activation of TLR3 attenuates the nigrostriatal degeneration in PD rats

**Figure 4:**
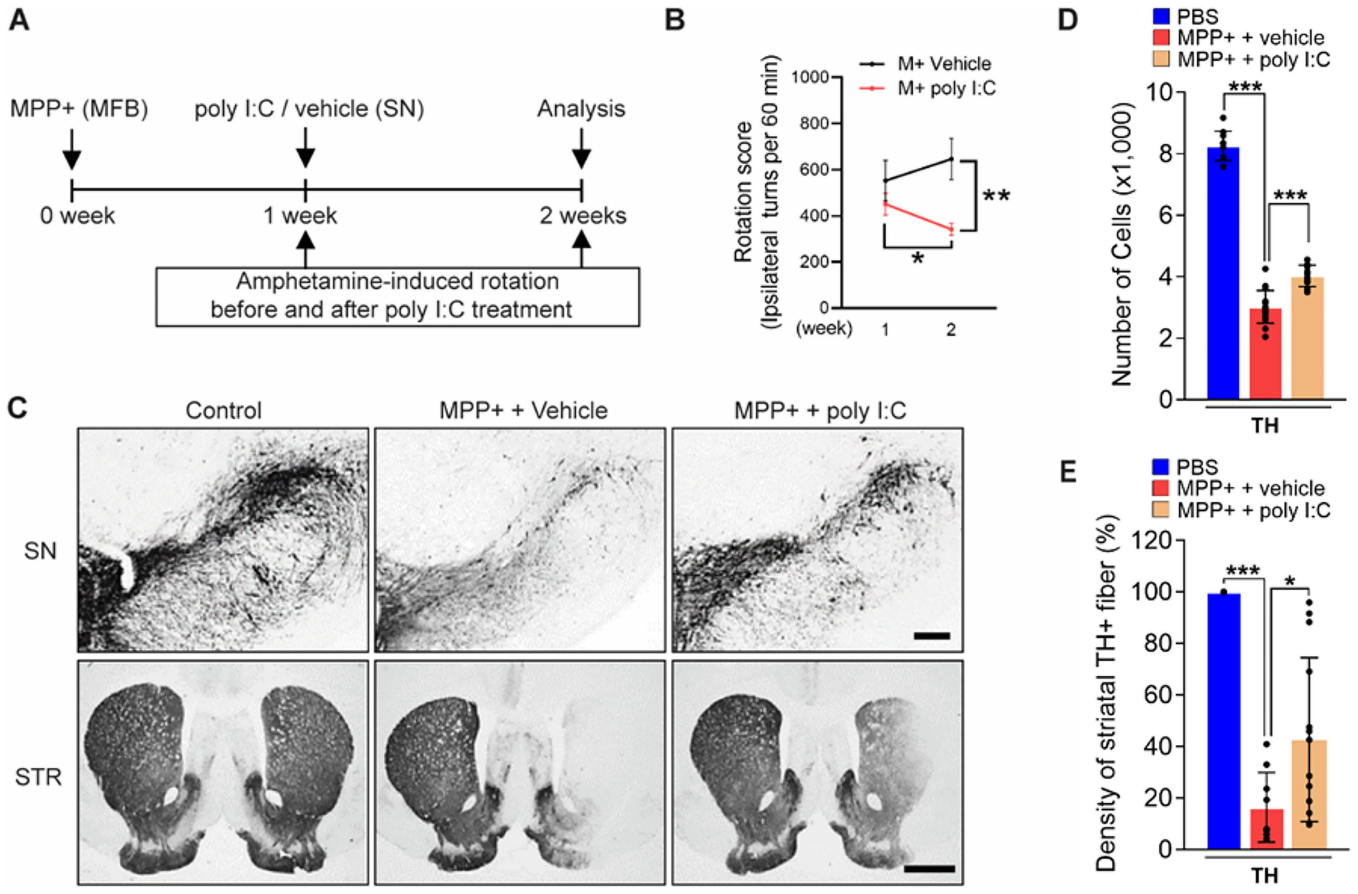
Poly I: C-activated astrocytic TLR3 exerts neuroprotection on DA neurons in PD. **(A)** Diagram of the experimental design. Rats received a unilateral injection of MPP^+^ into the MFB. All rats received poly I: C (4 μg/2 μL) or vehicle intranigrally in the SNpc 7 days after MPP^+^ injection and were transcardially perfused after the last rotation test. **(B)** The graph shows the number of amphetamine-induced rotations before (week 1) and after (week 2) poly I: C treatment compared to the vehicle in the MPP^+^-injected PD rats. **(C)** Representative immunohistochemical images show TH^+^ cells in the SNpc and TH^+^ fibers in the striatum (STR). Scale bars = 200 μm (SNpc), 2 mm (STR). **(D)** Bar graph shows the number of TH^+^ cells in the SNpc. **(E)** Bar graph shows the optical density of TH^+^ fibers in the striatum. ANOVA and Turkey’s multiple comparisons test **(B,D,E)**. *p < 0.05, **p < 0.01, ***p < 0.001. *n* = 7–13 in group (animals).

In parallel with showing neurotrophic astrocytes in the SNpc (Figure 3), we next tested whether MPP^+^-lesioned rats treated with poly I: C attenuate the neuronal death in the SNpc. Before investigating the pathology of the PD rat brain, we performed behavioral tests. We randomly and blindly divided the MPP^+^-lesioned rats that exhibited ipsilateral rotation behavior, as observed in the amphetamine-induced rotation test conducted 1 week after MPP^+^ administration (Figure 4A) (Nam et al., 2015; Kim et al., 2021). We subsequently injected poly I: C or vehicle. One week later (at 2 weeks), we performed the amphetamine-induced rotation test. Interestingly, the rats administered with poly I: C had a reduced number of amphetamine-induced ipsilateral rotations compared to the before poly I: C treatment (by 24.1%; 1 week vs. 2 weeks, *p* < 0.05) or vehicle treatment (by 47.1%; M + Vehicle *vs.* M + poly I: C, compared at week 2, *p* < 0.01) (Figure 4B). The data suggest that poly I: C attenuated progressive asymmetrical motor behavior. Next, the brains were harvested, and we analyzed whether endogenous TLR3 activation promotes the survival of DA neurons against progressive neurotoxicity caused by MPP^+^. The treatment with poly I: C significantly attenuated the MPP^+^-induced loss of nigrostriatal DA neurons, as measured by stereological counts of TH^+^ cells in the SNpc (Figures 4C,D) and density of TH^+^ fibers in the striatum (STR) (Figures 4C,E).

The expression of TLR3 in each cell type in the MPP^+^-injected PD rat model is replicated in the AAV2-α-syn-A53T-induced PD model, which overexpresses human A53T mutant α-syn (A53T) (Sato et al., 2011). At 3 weeks post-AAV2-α-syn-A53T injection, we observed an enhanced phosphorylation of α-syn (serine 129) as a marker of the pathological form of α-Syn in PD (Sato et al., 2011; Anderson et al., 2006), which indicates efficient transduction of α-syn into the DA neurons in the SNpc (Supplementary Figures S2A,B). Notably, in AAV2-α-Syn-A53T-injected rats, we observed increased TLR3 expression on GFAP+ astrocytes or OX-42 + microglia/macrophages compared to eGFP, while the expression of TLR3 on TH + DA neurons was decreased (Supplementary Figures S2C–F). In the treatment with poly I: C or a vehicle at 3 weeks post-AAV2-α-synuclein-A53T injection, poly I: C treatment significantly preserved the number of TH + cells in the SNpc at 4 weeks post-AAV2-α-synuclein-A53T injection compared to vehicle-treated rats (Supplementary Figures S2G,H). In accordance with the MPP^+^-induced PD rats, the findings suggest that TLR3, activated by poly I: C, alleviated the progressive neuronal death in PD rats.

Taken together, pharmacological activation of endogenous TLR3 on astrocytes induces NTF production, thereby attenuating DA neuron death and providing a disease-modifying strategy.

## Discussion

In the present study, we highlighted that TLR3 is primarily expressed in astrocytes in the nigral region of the human PD brain, as well as neurotoxin (MPP^+^)- or genetic alteration (AAV-α-Syn-A53T)-induced PD rat models. We observed that the poly I: C, an activator of TLR3, attenuates the degeneration of nigral DA neurons in the SNpc of the PD rat model. We also observed that poly I: C enhanced CNTF and VEGF-B production in astrocytes in the SNpc in PD rat brains. In short, our data strongly suggest that endosomal TLR3 activation, predominantly expressed in astrocytes in the SNpc, attenuates neuronal death in PD (Supplementary Figure S3). Our findings will help develop a potential therapeutic approach for inhibiting the progression of PD pathology.

The TLR family of innate immune receptors is mainly expressed in microglia or macrophages (Roodveldt et al., 2024). In the brains of individuals with Alzheimer’s disease (AD), the expression of TLR3 in microglia is elevated and associated with Aβ plaques (Walker et al., 2018). Against expectation, our results indicated that in the SNpc of MPP^+^-lesioned rats, TLR3 is expressed considerably on the GFAP+ cells, whereas no significant change in the expression level of TLR3 on microglia. In this context, we deeply considered the role of TLR3-expressing astrocytes in PD. In the literature, the effect of poly I: C-induced TLR3 activation on PD progression remains unclear. Activation of TLR3 has been implicated in neuroinflammation, leading to the loss of DA neurons in PD. Poly I: C induces vulnerability in DA neurons by stimulating astrocyte production of proinflammatory cytokines and chemokines (Park et al., 2006; Kim et al., 2008). Nonetheless, TLR3 activation by poly I: C enhanced antioxidative defense against oxidative stress in human fetal astrocytes (Borysiewicz et al., 2013). Furthermore, post-treatment of poly I: C reduced the infarct volume against cerebral I/R injury (Zhang et al., 2015). As part of the neuroprotective role of TLR3, our study first shows that poly I: C attenuates the loss of TH + cells in the SNpc and preserves striatal TH + fibers in MPP^+^-lesioned rats.

In post-mortem brains of PD patients, reactive astrocytes have been identified (Miklossy et al., 2006; Liddelow et al., 2017), and this is recapitulated in the SNpc of MPP^+^-lesioned rats. Subsequently, the astrocytes also expressed TLR3 in the SNpc of MPP^+^-lesioned rats. Although the TLR3 expression levels are not significantly upregulated by the MPP+, the sustained expression of TLR3 in astrocytes was amplified by the poly I: C. Notably, astrocytes express various NTF, including CNTF or VEGF-B, that attenuate or rescue the death of DA neurons in experimental rodent or culture PD models (Muller et al., 2007; Rothhammer et al., 2018; Nam et al., 2015; Yue et al., 2014; Falk et al., 2009). In this study, we observed that treatment with poly I: C to the MPP^+^-lesioned PD rats enhanced the expression of CNTF and VEGF-B, suggesting the endogenously expressed TRL3 on astrocytes in MPP^+^-lesioned rats are stimulated by the exogenous treatment with poly I: C. CNTF has been reported to be upregulated in GFAP+ astrocytes following lens injury in mice (Muller et al., 2007; Leibinger et al., 2009). Furthermore, GFAP+ astrocytes have been shown to express VEGF-B in the rat cortical cold injury model (Nag et al., 2002), and VEGF-B treatment promotes behavioral recovery and DA neuron preservation in PD models (Yue et al., 2014; Falk et al., 2011). Similarly, other NTFs such as brain-derived neurotrophic factor (BDNF) and mesencephalic astrocyte-derived neurotrophic factor (MANF), are upregulated in GFAP+ astrocytes following ischemic or spinal cord injuries (Bejot et al., 2011; Grade et al., 2013; Dougherty et al., 2000; Tokumine et al., 2003). Consistently, exogenous administration of these NTFs (CNTF, VEGF-B, BDNF, and MANF) has shown neuroprotective effects, ameliorated motor deficits, and prevented dopaminergic neuronal degeneration in toxin (6-OHDA, MPTP, and MPP^+^) -induced PD models (Yue et al., 2014; Aly et al., 2019; Hagg and Varon, 1993; Hao et al., 2017; Razgado-Hernandez et al., 2015; Revishchin et al., 2016). Moreover, we previously demonstrated that endogenous NTF production, specifically CNTF via astrocytic TRPV1 activation, protects dopaminergic neurons in a PD rat model (Nam et al., 2015). Taken together, these findings suggest that poly I: C treatment triggered the endogenous expression of both CNTF and VEGF-B, thereby revealing a novel mechanism of astrocyte-mediated NTF production that complements potential NTF-based therapies for PD. However, our study requires further testing to investigate the potential neuroprotective effect of poly I: C treatment on DA neuron death. Still, our study is limited to validating the mechanism underlying TLR3-mediated NTF production unless we test using an astrocyte-specific TLR3 knockdown system in a PD model.

While our findings highlight the therapeutic potential of TLR3 activation in PD models, translating these results into clinical settings requires a careful assessment of the systemic delivery and the treatment strategy to avoid potential adverse effects. Our pilot study using intravenous poly I: C administration does not recapitulate astrocyte-specific TLR3 activation (data not shown). Furthermore, earlier studies reported that systemic administration of poly I: C can induce viral challenges, potentially eliciting acute sickness behaviors or systemic inflammation (Field et al., 2010; Cunningham et al., 2007). The limitation of systemic administration of poly I: C is further compounded by the controversial effect of poly I: C in inducing inflammation vs. neuroprotection. Poly I: C treatment induces neuronal apoptosis and induces proinflammatory cytokines IL-1β, IL-6, or TNF-α in mice (Altahrawi et al., 2025; Qin and Crews, 2012; Okuma et al., 2023). Whereas systemic poly I: C treatment activates the neuroprotective pathway by alleviating amyloid β(1-42), reducing microglia and astrocytes activation, and decreasing pro-inflammatory mediators in the AD mouse brain (Zhu et al., 2025; Wang et al., 2023). The neuroprotective role of TLR3 is also tested by the TLR3 knock-out, which attenuates pressure-induced neuronal damage (Lin et al., 2024). Mechanically, the neuroprotection effect can be achieved by modulating autophagy to clear intracellular pathogens in macrophages (Delgado et al., 2008). Although the protective effect of poly I: C on PD pathology is not well established, our study displayed the potential treatment option to halt the progressive degeneration of DA neurons. However, the MPP^+^-lesioned rats we used in the study are limited in their characteristic to mimic the slow, multisystemic, and long-term progression of PD. Regarding AAV2-α-Syn-A53T rats, poly I: C-treatment failed to attenuate the loss of striatal TH + fibers or behavioral deficits (data not shown), although the death of TH + DA neurons was attenuated. Thus, further studies are needed using PD models that recapitulate the human PD pathology. Moreover, our studies should further aim to optimize poly I: C dosing regimens or employ a targeted delivery system to maximize the neuroprotective benefits while minimizing systemic inflammation.

In summary, we demonstrate that predominant expression of TLR3 in astrocytes is observed in the nigral lesion of the PD brain. Furthermore, TLR3 activation leads to the production of NTFs in astrocytes and attenuates DA neuron death in PD. Taken together, our data show that Poly I: C-activated TLR3 attenuates worsening PD pathology, suggesting that further research is needed to determine the therapeutic relevance of the current findings for PD.

## Data Availability

The original contributions presented in the study are included in the article/Supplementary material, further inquiries can be directed to the corresponding author. Statistical analysis was performed using GraphPad Prism Version 8.0.2, and GraphPad syntax used for the analysis is available from the corresponding author upon request.
