# Supplementary material for: TLR3 activation in astrocytes attenuates the nigrostriatal pathway degeneration in rodent models of Parkinson’s disease
Source: Front Cell Neurosci. 2026 Feb 4;20:1746731. doi: 10.3389/fncel.2026.1746731 (PMC12913188; doi:10.3389/fncel.2026.1746731)
Supplement: Supplementary file 1 [file Supplementary_file_1.docx]

Supplementary Material

TLR3 activation in astrocytes attenuates the nigrostriatal pathway degeneration in rodent models of Parkinson’s disease

Jaeyeong Jeong^1, 2^, So-Yoon Won^1^, Young Cheul Chung^3^, Won-Ho Shin^3, *^, Byung Kwan Jin^1, *^, Eun S. Park^4, 5, *^

^1^Department of Biochemistry & Molecular Biology, School of Medicine Kyung Hee University, Seoul 130-701, South Korea.

^2^Department of Molecular and Cellular Biology, Baylor College of Medicine, Houston, TX 77030, USA

^3^Department of Predictive Toxicology, Korea Institute of Toxicology, Daejeon 34114, South Korea.

^4^Vivian L. Smith Department of Neurosurgery, McGovern Medical School, The University of Texas Health Science Center at Houston, Houston, TX, USA.

^5^ Center for Neuroimmunology and Glial Biology, The Brown Foundation Institute of Molecular Medicine, The University of Texas Health Science Center at Houston, Houston, TX 77030, USA.

*** Correspondence:**Eun S. Park
Eunsu.park@uth.tmc.edu

Byung Kwan Jin
bkjin@khu.ac.kr

Won-Ho Shin
whshin@kitox.re.kr

| **Case No.** | **Final diagnosis** | **Age** | **Sex** | **PMD** | **Tissue** |
| --- | --- | --- | --- | --- | --- |
| 1 | Control | 79.6 | Male | 31.5 | SN |
| 2 |  | 69 | Male | 34 |  |
| 3 |  | 57 | Male | 48 |  |
|  |  |  |  |  |  |
| 1 | PD | 63.6 | Female | 56 | SN |
| 2 |  | 70 | Male | 32.5 |  |
| 3 |  | 72.1 | Male | 25 |  |
|  | | | | | |
| PMD, Postmortem delays (hour); SN, Substantia nigra | | | | |  |

**Supplementary Table 1. Human postmortem brain tissues were used for Figure 1.**

All human postmortem brain tissues were obtained from Victoria Brain Bank Network (VBBN) and delivered on the slide glass with paraffinization. Controls were not diagnosed with Parkinson’s disease and died of other medical causes, such as ischemic heart disease, coronary atherosclerosis, and metastatic malignant pleural mesothelioma. PMD, Postmortem delays (hours), SN, Substantia nigra.

**
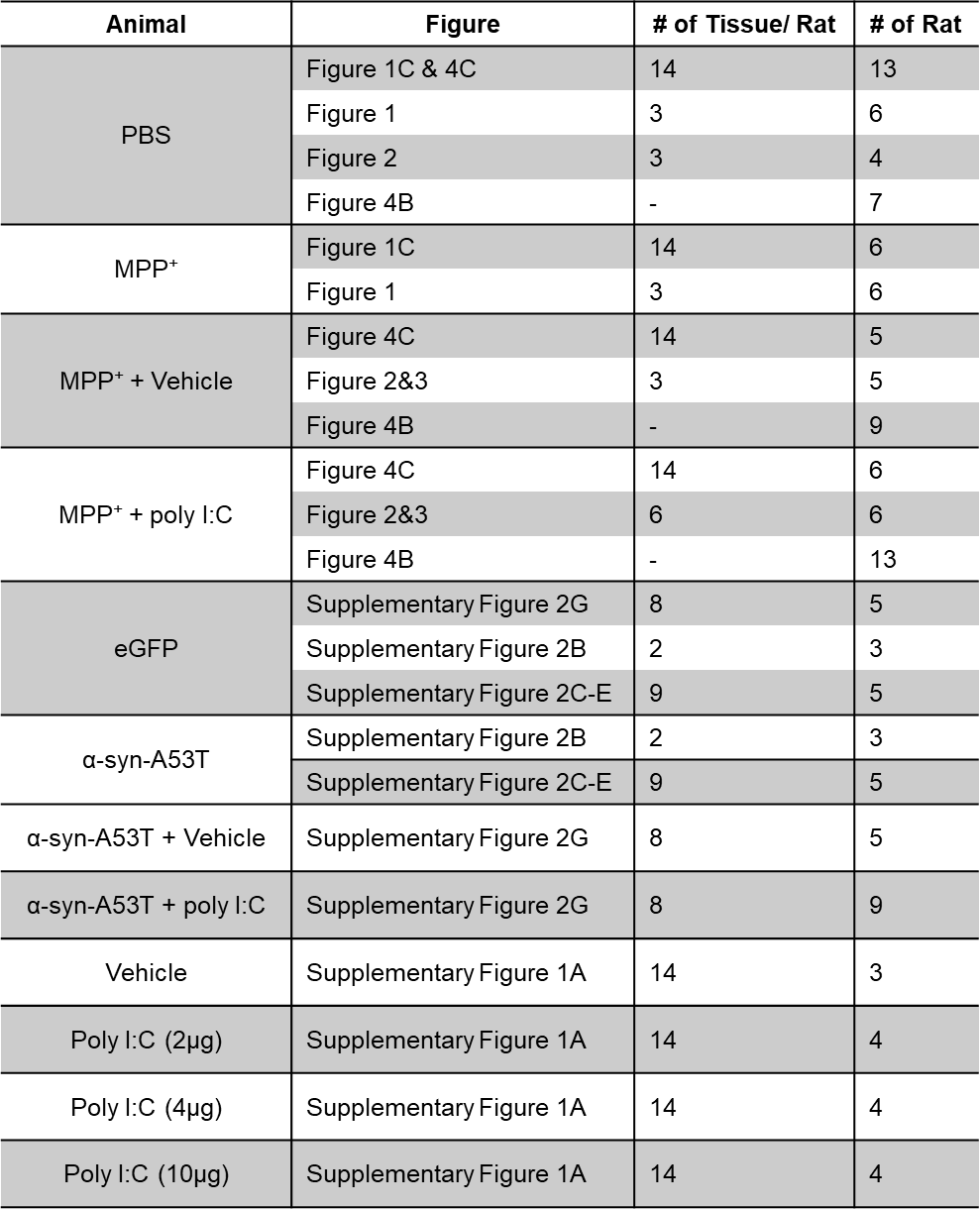
**

**Supplementary Table 2. Number of animals and tissues used for each analysis.**


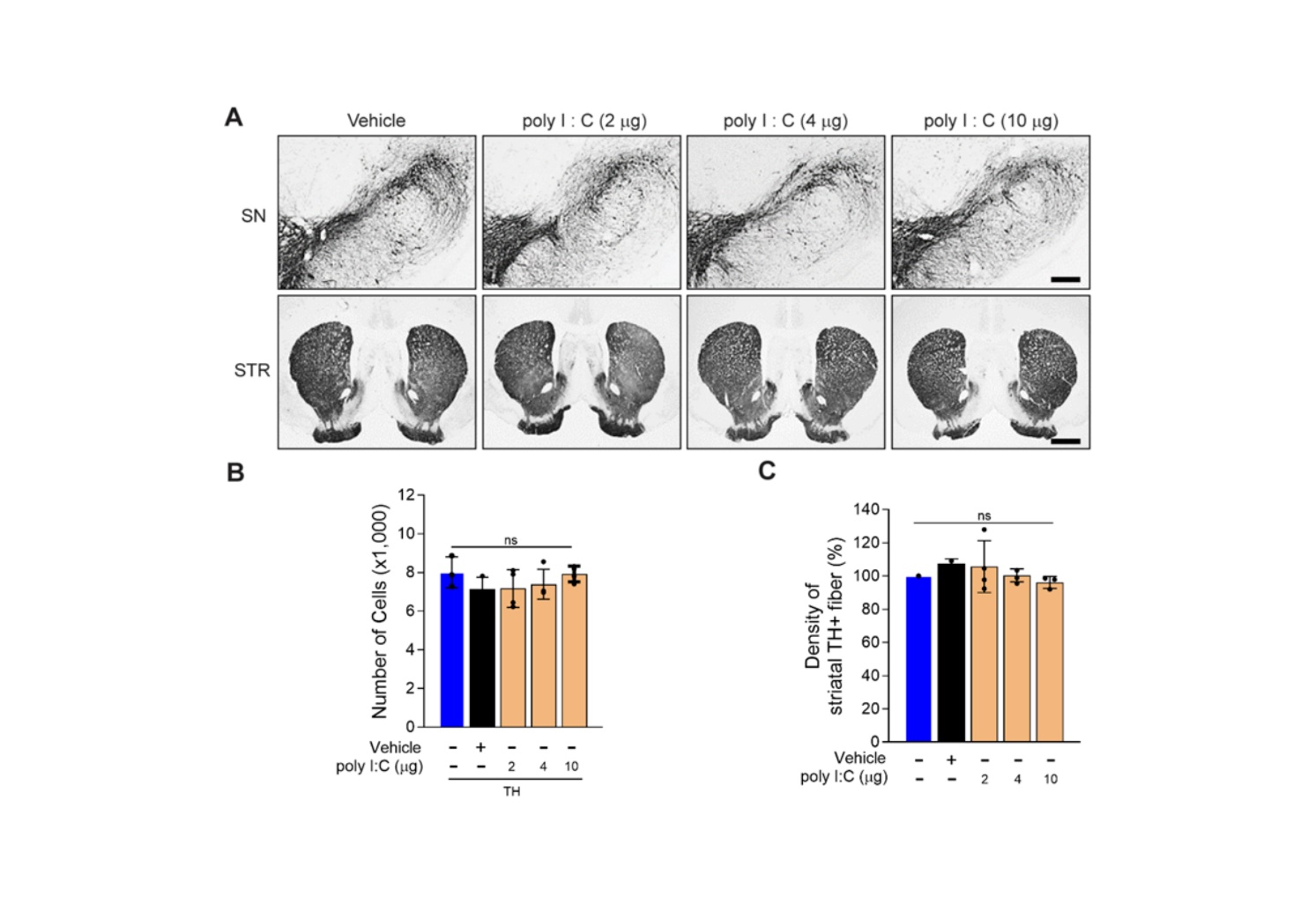


**Supplementary Figure 1. Intranigral injection of poly I:C has no toxicity on nigrostriatal dopamine neurons of intact rats.**

Various doses of poly I:C (2, 4, and 10 μg) were injected unilaterally into the SNpc of intact rats. Brain tissues were prepared for TH immunostaining at 1 week after poly I:C injection. (**A**) Representative photomicrograph shows the TH+ cells in the SNpc and TH+ fibers in the striatum (STR). Scale bars = 200 μm (SNpc), 2 mm (STR). (**B** and **C**) Bar graphs show the quantification of TH+ cells in the SNpc (B) and optical density of TH+ fibers in striatum (C). ANOVA and Turkey’s multiple comparisons test. ns, non-significant. Values are means ± SD. n = 3-4 in each group (animals).

**
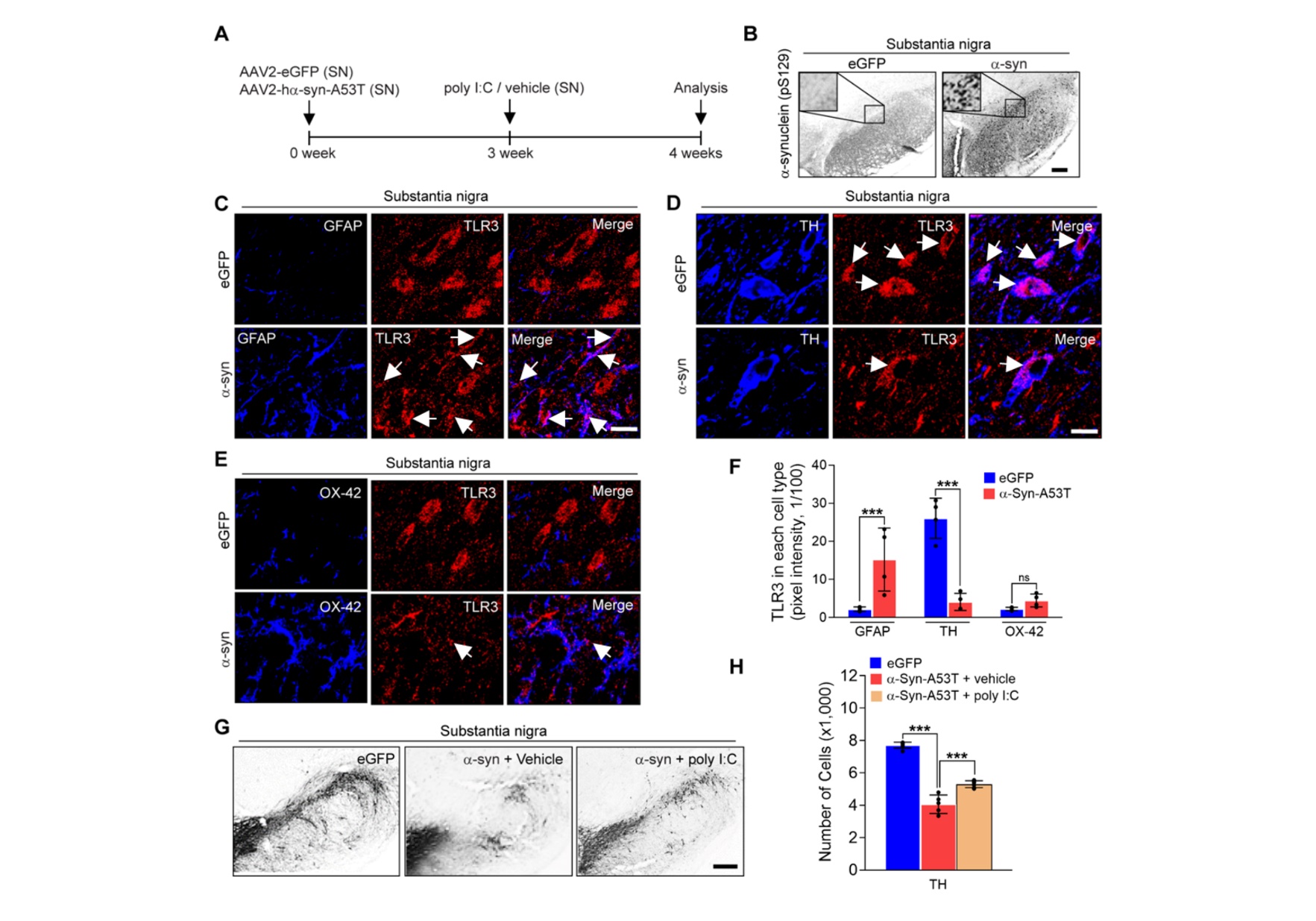
**

**Supplementary Figure 2. Poly I:C attenuates degeneration of DA neurons in the substantia nigra of α-synuclein-induced PD pathology.**

(**A**) Diagram of the experimental design. Rats received a unilateral injection of AAV2 human alpha-synuclein (α-syn)-A53T or AAV2-eGFP (control) into the SNpc. All rats received poly I:C (4 μg/2 μl) or vehicle intranigrally into the SNpc at 3 weeks after α-syn injection. At 1 week after poly I:C injection, rats were transcardially perfused, and brain tissues were prepared for immunohistochemical analysis. (**B**) Representative image shows the presence of phosphorylated α-syn in the SNpc 4 weeks after intra-nigral injection of AAV2-α-syn-A53T, compared to -eGFP. Scale bars = 200 μm. (**C**-E) Representative image shows the GFAP (blue; C), TH (blue; D), or OX-42 (blue; E), TLR3 (red; C-E), and merged (purple; arrow) in the SNpc of α-syn-A53T-lesioned PD rat brain. Scale bars = 20 μm. (**F**) Bar graph shows the quantification of TLR3 expression in each cell type. n=4 in each group (animals). (**G**) Representative image shows the TH+ cells in the SNpc. Scale bars = 200 μm. (**H**) Bar graph shows the number of TH+ cells in the SNpc. ANOVA and Turkey’s multiple comparisons test. ****p* < 0.001, ns, non-significant. Values are means ± SD. n = 5-9 in each group (animals).

**
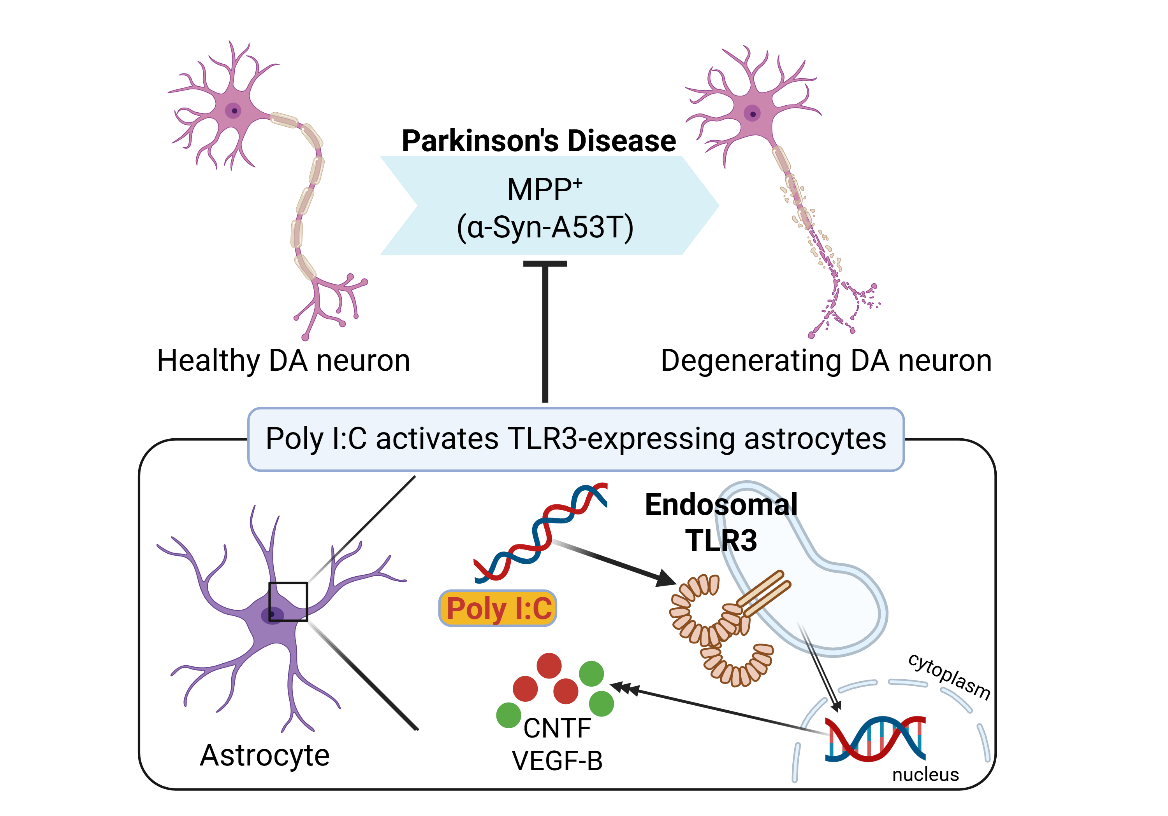
**

**Supplementary Figure 3. Poly I:C-activated TLR3 in astrocytes attenuates degeneration of dopaminergic neurons in a rat model of PD.** Administration of MPP^+^-(intra-median forebrain bundle) or AAV-α-syn-A53T-(intra-SNpc) induced degeneration of dopamine (DA) neurons in the PD rat model. Human PD brains and PD rats display TLR3 expression in astrocytes in the SNpc. Intra-nigral (SNpc) injection of Poly I:C activates endosomal TLR3 in astrocytes and thereby astrocytes produce NTFs such as CNTF and VEGF-B. Abbreviations: MPP^+^, 1-methyl-4-phenylpyridinium; AAV, Adeno-associated Virus; α-syn, alpha-synuclein; TLR3, Toll-like receptor 3; NTF, neurotrophic factor; CNTF, ciliary neurotrophic factor; VEGF-B, vascular endothelial growth factor. *Created in BioRender. Park, E. (2026) https://BioRender.com/d32t564*
